# Dapsone in dermatology and beyond

**DOI:** 10.1007/s00403-013-1409-7

**Published:** 2013-12-06

**Authors:** Gottfried Wozel, Christian Blasum

**Affiliations:** 1 Study Centre for Clinical Trials, Dermatology, Gesellschaft für Wissens- und Technologietransfer der Technischen Universität Dresden mbH, Blasewitzer Str. 43, 01307 Dresden, Germany; 2 Private Practice of Dermatology, Marktplatz 25, 73728 Esslingen, Germany

**Keywords:** Dapsone, Antiinflammatory mechanisms of action, Adverse effects, Therapeutic strategies, Use in dermatology, Non-dermatological disorders

## Abstract

Dapsone (4,4′-diaminodiphenylsulfone) is an aniline derivative belonging to the group of synthetic sulfones. In 1937 against the background of sulfonamide era the microbial activity of dapsone has been discovered. Shortly thereafter, the use of dapsone to treat non-pathogen-caused diseases revealed alternate antiinflammatory mechanisms that initially were elucidated by inflammatory animal models. Thus, dapsone clearly has dual functions of both: antimicrobial/antiprotozoal effects and anti-inflammatory features similarly to non-steroidal anti-inflammatory drugs. The latter capabilities primarily were used in treating chronic inflammatory disorders. Dapsone has been investigated predominantly by in vitro methods aiming to get more insights into the effect of dapsone to inflammatory effector cells, cytokines, and/or mediators, such as cellular toxic oxygen metabolism, myoloperoxidase-/halogenid system, adhesion molecules, chemotaxis, membrane-associated phospholipids, prostaglandins, leukotrienes, interleukin-8, tumor necrosis factor α, lymphocyte functions, and tumor growth. Moreover, attention has been paid to mechanisms by which dapsone mediates effects in more complex settings like impact of lifespan, stroke, glioblastoma, or as anticonvulsive agent. Additionally, there are some dermatological investigations in human being using dapsone and its metabolites (e.g., leukotriene B_4_-induced chemotaxis, ultraviolet-induced erythema). It could be established that dapsone metabolites by their own have anti-inflammatory properties. Pharmacology and mechanisms of action are determining factors for clinical use of dapsone chiefly in neutrophilic and/or eosinophilic dermatoses and in chronic disorders outside the field of dermatology. The steroid-sparing effect of dapsone is useful for numerous clinical entities. Future avenues of investigations will provide more information on this fascinating and essential agent.

## Introduction

Dapsone was first synthesized in 1908 [63]. At that time, dapsone was not envisioned as a therapeutic agent, but was the result of pure chemical science ambition. Sulfone research in medicine started in 1937, when two groups in England and France were the first to investigate dapsone as an antimicrobial agent in the framework of sulfonamide research [22, 43, 59]. In this context, it is remarkable that certain congeners, but not the so-called parent sulfone dapsone, were the first sulfones used to treat gonorrhea [18, 123]. Later, introduction into the therapy of non-infectious diseases was not the result of systematic research, but virtually driven by fortune [170, 173].

Concerning the mechanisms of action, dapsone is characterized by dual function. It combines both antimicrobial/antiprotozoal properties and anti-inflammatory effects resembling those of non-steroidal anti-inflammatory drugs. In past decades, especially the hematological adverse effects of dapsone have induced intense efforts to develop substituted sulfones with improved risk–benefit-ratio. A parallel goal of research was the synthesis of parenteral application modalities. Interestingly, however, still today the oral application of dapsone constitutes the only sulfone used in systemic therapy.

The changeful history of dapsone has been covered in detail in the literature [47, 165, 170]. Therefore, this issue has been omitted from this survey. Rather, this paper will focus on the mechanisms of action of dapsone and consider important indications in dermatology and beyond as well as therapeutic consequences. In order to understand the mechanisms of action of sulfone and its adverse effects, knowledge of its pharmacology and metabolism in human beings and in inflammatory cells is mandatory.

## Chemistry and pharmacology

**Fig. 1:**
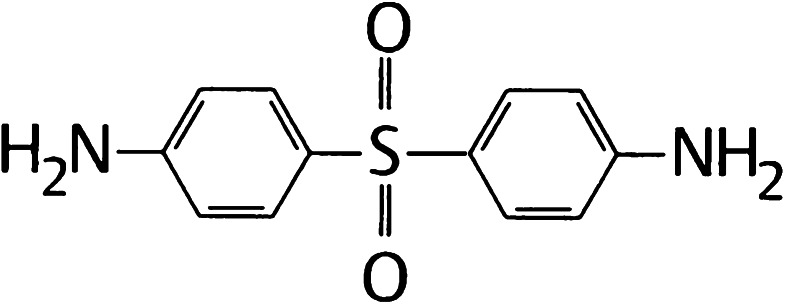
Structural formula of dapsone (4,4′diaminodiphenylsulfone)

**Fig. 2:**
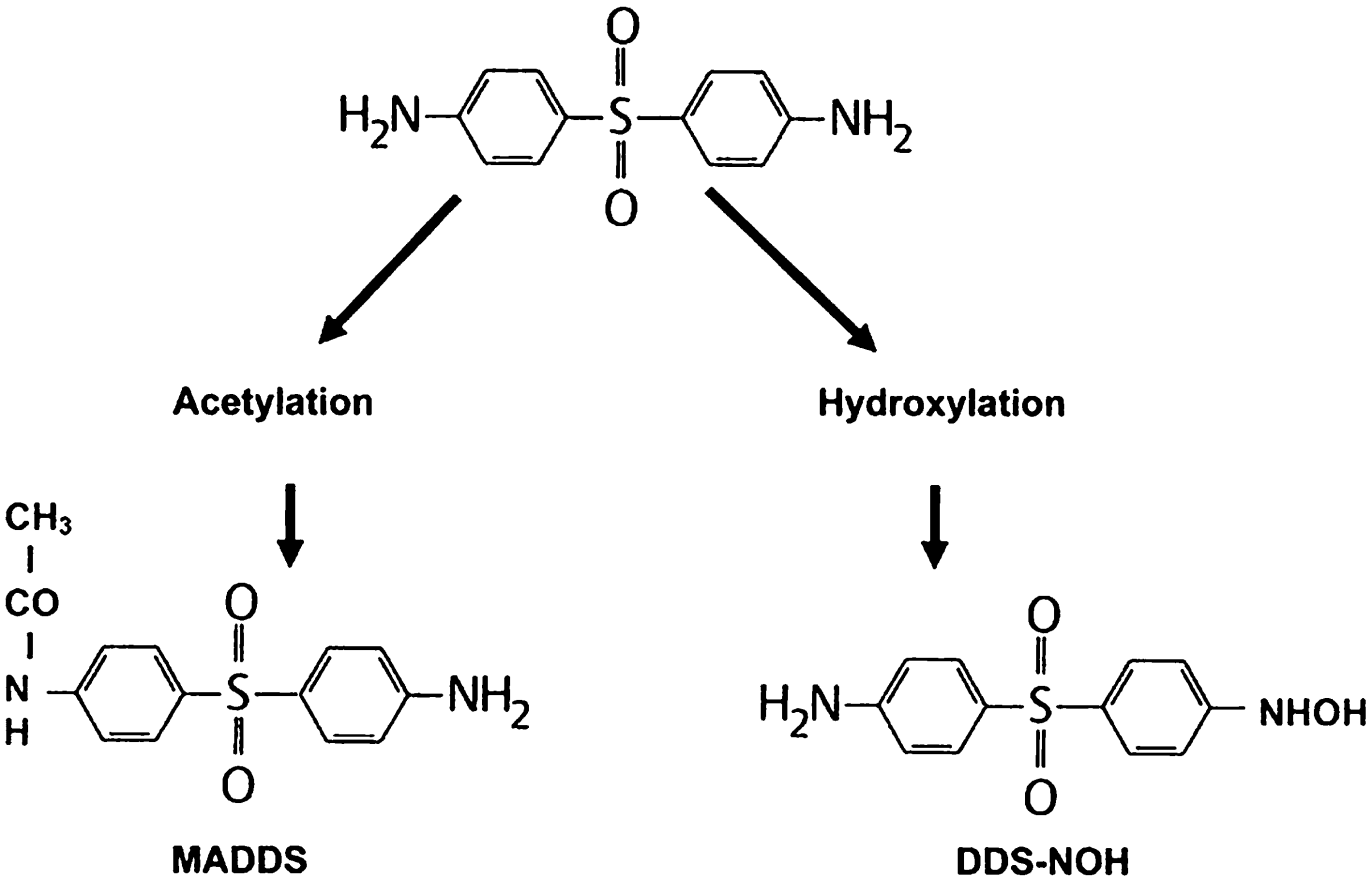
The two major metabolic pathways of dapsone (*MADDS* monoacetyldapsone, *DDS-NOH* dapsone hydroxylamine)

Chemically, dapsone is an aniline derivative. As a sulfone, it shows the structure of a sulphur atom linking to two carbon atoms (Fig. 1). Solubility of dapsone varies over a wide range depending on the solvent used (e.g. water, 0.2 mg/mL vs. methanol, 52 mg/mL). Following oral administration, dapsone is almost completely absorbed from the gut with bioavailability exceeding 86 %. Peak serum concentrations are attained within 2–8 h. After ingestion of a single 50–300 mg dose of dapsone, maximum serum concentrations range from 0.63 to 4.82 mg/L [2, 165, 181]. Under steady-state conditions, 100 mg/day (the dose most frequently used) results in serum concentrations of 3.26 mg/L (maximum) and 1.95 mg/L (after 24 h) [2, 41, 181]. These dapsone serum concentrations attained in vivo must be kept in mind when interpreting the results of in vitro investigations (see below). After absorption, dapsone undergoes enterohepatic circulation. It is metabolized by the liver but also by activated polymorphonuclear leukocytes (PMN) and mononuclear cells [152, 156]. In the liver, dapsone is metabolized primarily through acetylation by *N*-acetyltransferase to monoacteyldapsone (MADDS), and through hydroxylation by cytochrome P-450 enzymes, resulting in the generation of dapsone hydroxylamine (DDS-NOH) (Fig. 2). In fact, administration of dapsone has been utilized to determine the acetylation phenotype (rapid vs. slow acetylator). In terms of both efficacy and induction of adverse effects, the most important issue is the generation of DDS-NOH. This metabolic pathway also occurs in lesional skin of inflammatory dermatoses and is thought to be mediated by activated PMN [156]. Dapsone is distributed to virtually all organs. Dapsone is retained in skin, muscle, kidneys, and liver. Trace concentrations of the drug may be presented in these tissues up to 3 weeks after discontinuation of dapsone treatment. The drug is also distributed into sweat, saliva, sputum, tears, and bile. Dapsone is 50–90 % bound to plasma proteins, whereas MADDS is almost completely bound to plasma proteins. It crosses the blood–brain barrier and placenta and is detectable in breast milk [20, 137]. Cases have been reported where dapsone therapy of the mother resulted in neonatal haemolysis and cyanosis [105]. Approximately 20 % of dapsone is excreted as unchanged drug via urine, whereas 70–85 % is eliminated as water-soluble metabolites after conjugation with glucuronic acid. This step is mediated by uridine diphosphate (UDP)-glucuronosyltransferase. Additionally, a small amount might be excreted in faeces including some yet unidentified metabolites. The complex metabolic pathway of dapsone has been reviewed in detail several times [156, 159, 164, 165, 178, 181].

**Fig. 3:**
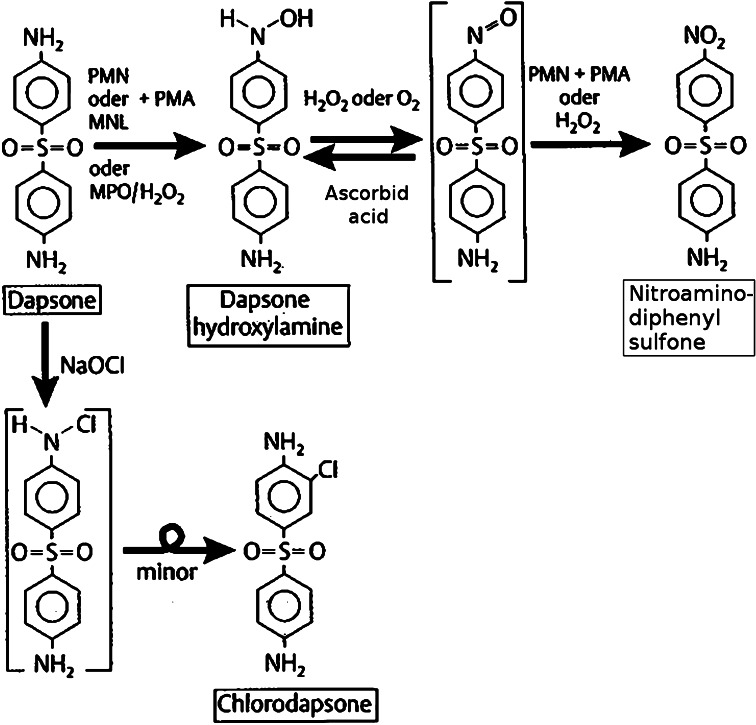
Dapsone metabolism in human PMN and mononuclear cells after activation by phorbol myristate acetate (PMA) and oxidation path by NaOCl (according to Uetrecht et al. [156])

Metabolism of dapsone in cell cultures has not been studied as extensively [17]. In part, this can be attributed to the chemical properties of dapsone, which make it a difficult-to-handle compound [165]. First findings concerning the metabolism of dapsone in cell cultures were presented by Drayer et al. [49] and the Canadian group of Uetrecht et al. [156]. Following incubation of PMN- and zymosan-activated human PMN with dapsone, high-pressure liquid chromatography and gas chromatography/mass spectroscopy demonstrated the production of dapsone hydroxylamine (–NO_2_), (Fig. 9) and a chlorine-substituted derivative of dapsone (–Cl) (chlorodapsone) (Fig. 10). Without prior stimulation, neither DDS-NOH nor the nitro derivative were detectable. The authors postulate the biotransformation as depicted in Fig. 3. Activation of leukocytes results in the induction of the respiratory burst pathway with consecutive production of reactive oxygen-species (ROS) such as ^1^O_2_ (singlet-O_2_), H_2_O_2_ or OH^−^. During this process, myeloperoxidase (MPO) uses dapsone as substrate resulting in the generation of DDS-NOH via oxidation. Finally, by a further non-enzymatic oxidation process, the nitro derivative of dapsone is generated (Fig. 11).

Controls in cell-free settings using purified myeloperoxidase and H_2_O_2_ confirmed these findings. In contrast, adding catalase or sodium azide, respectively, led to a dose-dependent inhibition of the oxidation of dapsone. The authors were not able to demonstrate a presumptive alternative enzymatic pathway of hydroxylation of dapsone through the prostaglandin pathway, as classic inhibitors like acetylsalicylic acid or indometacin did not induce a decrease of oxidation. Dapsone metabolism in human mononuclear cells has been demonstrated to be quite similar [156].

When dapsone is administered, there is equilibrium between acetylation and deacetylation. Thus, there is the possibility that PMN in peripheral blood are exposed both to dapsone and its metabolites. These metabolites like DDS-NOH have been shown to be pharmacologically active. However, they have been made responsible not only for anti-inflammatory mechanisms (e.g., inhibition of chemotaxis) but also for a number of side effects. To date, this has been clearly documented for DDS-NOH [35]. Interestingly, Khan et al. [95] recently demonstrated that human keratinocytes which had been stimulated by various cytokines like tumor necrosis factor α (TNF-α), interleukin 1β (Il-1β), and interferon γ (INF-γ) can produce DDS-NOH as well.

## Antimicrobial activity

As an antimicrobial agent, dapsone is bacteriostatic in action. It inhibits the synthesis of dihydrofolic acid through by competing with para-aminobenzoic acid for the active site of dihydropteroate synthetase [35, 41], thus resembling the action of sulphonamides. Sulfones were found to suppress the growth of various pathogenic bacteria such as streptococci, staphylococci, pneumococci, mycobacteria, and other strains. The mechanism of action of topical dapsone in the treatment of acne vulgaris may result from a combination of both antiinflammatory and antimicrobial effects. In vitro, dapsone has some antibacterial activity against *Propionibacterium* *acnes*. Owing to its antimicrobial activities, dapsone is clearly playing a role in the treatment of certain infectious diseases (see section “Indications”) [67].

## Anti-inflammatory mechanisms of action

### Animal studies

In the 1970s, dapsone was studied in numerous inflammation models in animals. Experiments were conducted especially with small mammals like mice, rats and guinea pigs (Table 1). Results of these studies showed wide variations in anti-inflammatory efficacy. Whereas some authors were able to demonstrate pronounced anti-inflammatory activities (e.g. in anthralin-induced mouse ear swelling test (MEST), others failed to do so (e.g. in arachidonic acid-induced MEST). Gemmel et al. [64] and Lewis et al. [102], for example, showed that the antiinflammatory action of dapsone differs from that of aspirin-like non-steroidal anti-inflammatory drugs (NSAID’s).

**Table 1:** Investigations of dapsone in common animal inflammatory models

Model	Dose range	Authors	References
A single dose studies	mg/kg		
Carrageenan-oedema (rat)	36–160	Capstick and Lewis	[23]
Kaolin-induced oedema (rat)	11–100	Lewis et al.	[102]
Zymosan-induced oedema (rat)	10–100	Gemmel et al.	[64]
Reverse passive Arthus-type reaction (rat)	10–100		
UV erythema (guinea pig)	ED_50_–160	Lewis et al.	[102]
B multiple dose studies	mg/kg/day		
Reverse passive Arthus-type reaction (guinea pig)	2–5	Ruzicka et al.	[131]
Aktive Arthus-type reaktion (guinea pig)	5–50	Thompson and Souhami	[150]
Cotton wool pellet granuloma (rat)	200	Capstick and Lewis	[23]
Carrageenan-impregnated	50–200	Lewis et al.	[102]
Cotton wool pellet granuloma (rat)			
Adjuvant arthritis (rat)	100–200	Capstick and Lewis	[23]
		Lewis et al.	[102]
Hypervitaminosis A (rabbit)	25	Barranco	[8]
Arachidonic acid-induced mouse ear swelling test (AA-MEST)	0.1; 1; 5; 10 %	Christoph	[29]
Anthralin-induced MEST	Topically and systemically	Salomon	[135]

Hence, it is difficult to draw reliable conclusions regarding the mechanisms of action of dapsone in sulfone-sensitive diseases. In this respect, several factors have to be considered. Amongst others, the inflammation models used are only poorly characterised on phenotypic and genotypic levels. Often, dapsone-concentrations applied were considerably exceeding those achieved under therapy. Apart from that, single dose application cannot be readily compared with repeated doses. Finally, testing conditions varied substantially (e.g. pre-therapeutic vs. post-therapeutic application of the sulfone). Despite these limitations, dapsone can be attributed an anti-inflammatory potential that—cum grano salis—equals that of classic NSAID’s. In the following, the impact of dapsone on isolated proinflammatory signal transduction pathways and on more complex disease processes is described in detail.

### Reactive oxygen species

**Fig. 4:**
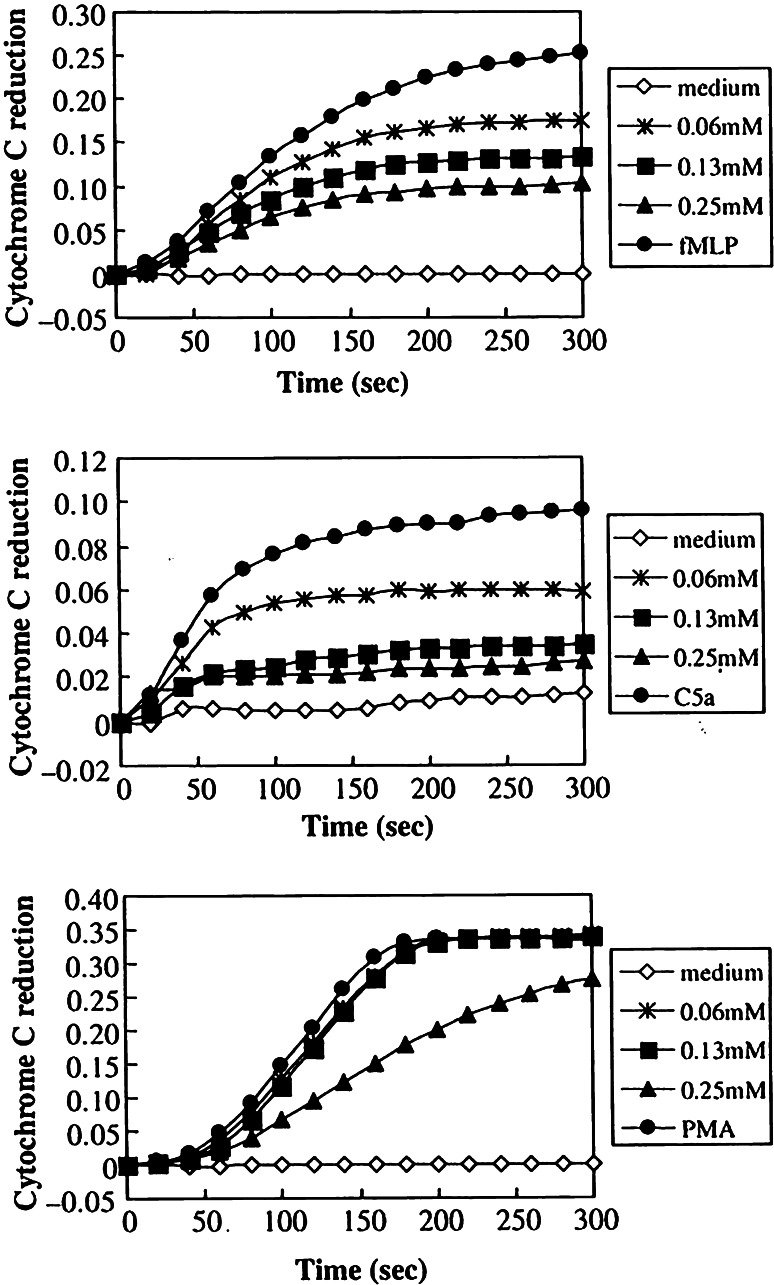
The effect of dapsone on the extracellular superoxide (O_2_^−^) release induced by *N*-formyl-l-methionyl-l-leucyl-l-phenyalanine (fMLP), C5a and phorbol myristate acetate (PMA). Neutrophils (1 × 10^6^ cells mL^−1^) were preincubated with or without dapsone at the concentrations indicated for 30 min and then stimulated with fMLP (1 μmol L^−1^) (**a**), C5a (100 nmol L^−1^) (**b**) or PMA (100 nmol L^−1^) (**c**). After addition of cytochrome C (20 μmol L^−1^), the reduction of absorbance at 550 nm in the absence (ΔOD_550_[SOD]) was measured for 5 min using a single-beam spectrophotometer. SOD-inhibitable cytochrome C reduction corresponded to ΔOD_550_–ΔOD_550_[SOD]. The data shown are representative of three to four separate experiments with similar results. Dapsone suppressed the extracellular O_2_^−^ production induced by fMLP and C5a but not by PMA. (according to Suda et al. [148])

**Fig. 5:**
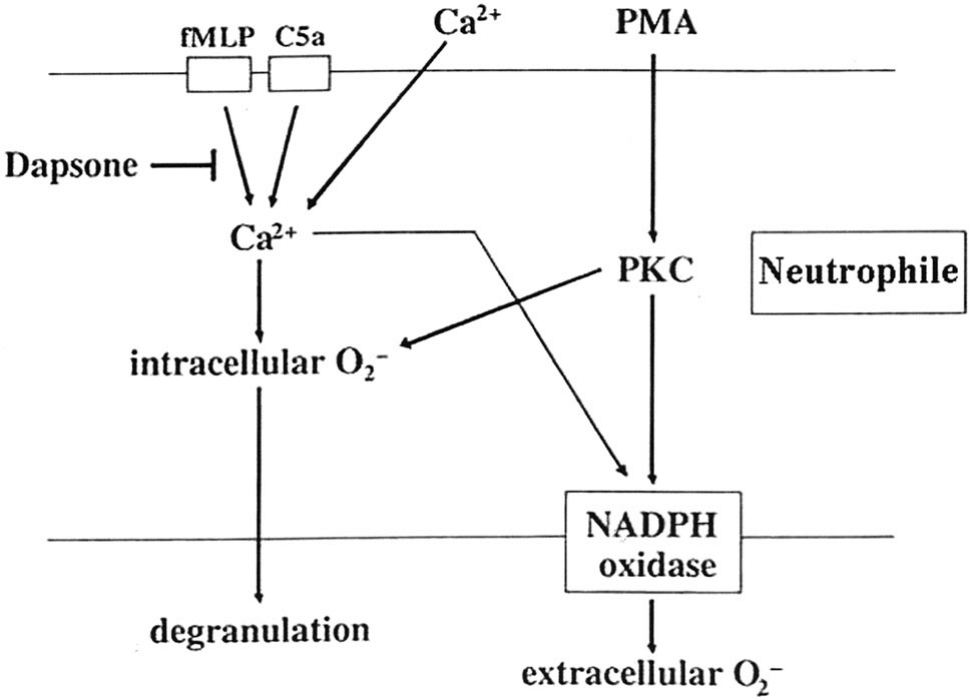
The effect of dapsone on neutrophils. Dapsone suppressed intra- and extracellular production of superoxide (O_2_^−^) and elastase release triggered by FLMP and physiological agonist C5a, but not by PMA. Both FMLP and C5a signaled the above pathways by inducing calcium influx, but PMA functions bypassed calcium influx. Dapsone was capable of antagonizing the induction of calcium influx (FMLP: *N*-formyl-l-methionyl-l-leucyl-l-phenyalanine, *PMA* phorbol myristate acetate, *PKC* protein kinase C, *NADPH* Nicotinamide adenosine dinucleotide phosphate) (according to Suda et al. [148]

Historically, Japanese authors were the first in 1983 to attribute an oxygen-radical scavenging ability to dapsone [112–115, 121, 161]. In the works of this group, dapsone was shown to effectively lower the concentrations of H_2_O_2_ and of OH^−^ as well as the activity of chemiluminescence when these parameters were studied during the process of oxidative activation PMN. However, dapsone did not alter the concentration of O_2_^−^. Experiments by van Baar et al. [unpublished data] confirmed these findings. Furthermore, the Japanese authors were able to demonstrate an effect of dapsone to reduce the concentrations of extracellular ROS as well as that of singulett ¹O_2_. In these experiments the xanthine/xanthine oxidase system had been tested in the absence of both PMN and superoxide dismutase (SOD). Accordingly, the ROS—but not O_2_^−^-lowering effect of dapsone, is unlikely to be mediated by an interaction of dapsone and SOD. In contrast to these conclusions it was claimed by later publications that dapsone actually suppressed *N*-formyl-methionyl-leucyl-phenylalanine (FLMP)-induced production of extracellular O_2_^−^ [43]. More recent studies by Suda et al. [148] yielded comparable data with those of Debol et al. [43]. Suda et al. utilized human PMN, stimulated by FLMP, C5a and PMA and evaluated any influence of dapsone on the generation of extracellular O_2_^−^ (using a cytochrome C reductase assay), intracellular O_2_^−^ (using flow cytometry), the generation of elastase and on cytosolic free ionized calcium. They found dapsone to cause dose-dependant, sizeable reductions of intracellular O_2_^−^ (after induction by FLMP and C5a) and of extracellular O_2_^−^ (Fig. 4a–c). Stikingly, in these experiments, dapsone appeared to exert little or no effect on PMA-stimulated O_2_^−^ production. The authors demonstrated an influx of calcium into cells in response to FLMP. In contrast, PMA failed to mobilize calcium. Dapsone was seen to inhibit calcium influx in response to FLMP or C5a. Similarly, dapsone was able to inhibit any generation of elastase in the presence of FLMP and C5a. Given that PMA failed to cause an influx of calcium, one may conclude that dapsone exerts some direct effect on the intracellular concentrations of free ionized calcium when the latter was induced by signalling mechanisms [148]. With respect to the O_2_^−^ production and the effects of dapsone in PMN the authors suggested a pathway which is shown in Fig. 5. The FLMP-induced calcium influx in PMN and its suppression by dapsone were confirmed by other laboratories [43].

The role of dapsone in the generation of reactive oxygen species (ROS) has also been studied in non-phagocytizing cells [27]. These authors utilized diploid fibroblasts obtained from human newborns. The cells were stimulated by paraquat, a herbicide. Paraquat is a known inducer of oxidative stress. Its effect involves the membrane-bound NADPH oxidase and the production of superoxide anion. The intracellular mitochondrial complex may also participate in this process. In the experiments, dapsone—applied in different concentrations—leads to a reduction of the expression of NADPH oxidase 4. Cho et al. [27] found an inhibition of paraquat-inducible proteinkinase C, of the mitochondrial complex, and they showed a reduced production of cytosolic and mitochondrial superoxide anion. Moreover, they showed that dapsone as parent compound does not possess a scavenger function for ROS. In summary, results of these studies repeatedly disclosed that dapsone possesses substantial antioxidative properties that can be attributed in large parts to an inhibition of ROS production. This calls earlier studies into question that had ascribed dapsone with direct scavenger function [169]. Apart from this, this experimental study shows for the first time that experimentally stimulated cells without phagocytic properties are capable of producing ROS. In another experiment, the same group examined the mitochondrial complex V of *Caenorhabditis elegans* (*C. elegans*) and detected a drop in protein levels in dapsone-treated animals. Again, dapsone inhibited paraquat-induced release of H_2_O_2_. Finally, the authors could reproduce the same effect in mammalian muscle cells (male BALB/c mice and C2C12 cells) [26]. These findings confirmed that not only stimulated inflammatory cells can generate ROS that are being down regulated by dapsone.

### Myeloperoxidase/halide system

#### MPO mediates the transformation of H_2_O_2_ via halogenation

$$ {\text{H}}_{2} {\text{O}}_{2} + {\text{ Cl}}^{ - } \underrightarrow{\text{myeloperoxidase}}\, {\text{H}}_{2} {\text{O}} + {\text{ ClO}}^{ - } $$

In a further reaction, ClO^−^ and H_2_O_2_ can produce the release of singlet-O_2_ and thereby amplify the cytotoxic potential. It has been generally agreed that part of the anti-inflammatory activity of dapsone results from a (reversible) inhibition of MPO. This leads to an inhibition of the conversion of H_2_O_2_ to HOCL. Early works on the mechanisms of action had already postulated a direct inhibition of MPO by dapsone [144, 161]. Subsequent studies have confirmed the inhibitory effect of dapsone on MPO [19, 88, 92, 93, 157]. It is assumed that dapsone exerts a direct inhibition of MPO which leads to the formation of inactive intermediates of the enzyme. As hypochlorous acid is an integral part not only of the antibacterial/antiprotozoal armamentarium of PMN and eosinophils but can also cause tissue damage in non-infectious disease states [163], the inhibition of MPO by dapsone might account for the anti-inflammatory potential of the sulfone.

### Adhesion molecules/cellular adhesion

Leukocyte function antigen (LFA-1) (CD 11 a/CD 18), Mac-1 (CD 11b/CD 18) and p 150, 95 (CD 11 c/CD 18) are important members of the β_2_-integrin family of adhesion molecules. The effects of dapsone on Mac-1-mediated adherence of neutrophils of healthy persons were examined by Booth et al. [16]. They observed a dose-dependent inhibition of adherence, but only when cells were stimulated with either FMLP or PMA. Adherence of unstimulated cells remained unchanged under the influence of dapsone. Addition of integrin- β_2_-antibodies proved that adherence was indeed integrin-mediated. Yet it is not clear whether these findings are of relevance in patients treated with dapsone as the concentrations used exceed those under therapy by a factor of 10–100. Heidenbluth et al. [unpublished data] confirmed the ability of dapsone to interfere with the function of integrins. They examined the expression of leukocyte integrins LFA1, Mac-1 and p 150, 95 in various cell populations under the influence of dapsone (20, 100 and 200 μg/mL). Fluorescence–activated cell sorter (FACS) analysis was performed in human whole blood, in isolated PMN and in the macrophage cell line U937. Stimulation with PMA induced an increased expression of CD11 a, b, c and CD18 both in PMN and in U937 cells. Dapsone inhibited the expression of β2-integrins CD11a, CD11c and CD18 in PMN only marginally, but significantly in U937-cells (CD11c). This effect was not dose dependent. Thuong-Nguyen et al. [151] showed that dapsone inhibited adherence of neutrophils to IgA and IgG from sera of patients with IgA-linear dermatosis and bullous pemphigoid in a dose-dependent manner. Other investigators established an inhibition of adhesion of TNF-α- or platelet activating factor (PAF)-activated PMN of healthy donors by dapsone [116]. This group used a frozen section adhesion assay of healthy skin under incubation with IFN-γ and observed an inhibition of neutrophil adhesion to epidermal cells in a concentration range from 0.1 to 80 μg/mL dapsone. They could also verify that dapsone down regulates the expression of CD11b on activated neutrophils.

### α-1-Protease inhibitor

In vitro, dapsone possesses a protective effect on elastase inhibitor capacity of α-1-protease inhibitor (AIP), thereby showing similar properties as the physiologic antioxidant ceruloplasmine. Once more, this AIP-protective effect has been linked to the potent suppression of ROS-production and to the inhibition of the MPO-halide-system [149]. As the dapsone concentrations used in this study resemble that attained in vivo, this action might be useful in the therapy of diseases with AIP dysfunction.

### Chemotaxis

The impact of dapsone on chemotaxis has been studied with partly conflicting results. A pilot study utilized various chemotaxins and found a selective inhibition by dapsone in concentrations resembling those under therapy only when FMLP was used as stimulus, but not when leukocyte-derived chemotactic factor (LDCT) and C5a were applied [72]. Moreover, species- and cell dependency could be observed; PMN of guinea pigs and monocytes did not show inhibition of chemotaxis. Booth et al. [16] examined chemotaxis and random migration by agarose-technique using FMLP and IL-8 as stimuli. Dapsone was added in concentrations of 30, 100 and 300 μg/mL. Again, a dose-dependent inhibitory effect of dapsone on chemotaxis of human neutrophils could be observed (FMLP: 30 μg dapsone/mL: 24 % inhibition, 300 μg dapsone/mL: 62 % inhibition, IL-8: 100 μg dapsone/mL: 24 % inhibition, 300 μg dapsone/mL: 61 % inhibition). Once more, random migration was also inhibited by dapsone. In vivo studies produced inconsistent results. A short-term study with rabbits and guinea pigs which were fed dapsone for 3 days showed no influence of dapsone on chemotaxis of PMN [131]. A1h *E. coli* activated autologous serum even showed that dapsone induces an increase in chemotaxis in healthy individuals and in leprosy patients [6]. In a human in vivo study in healthy volunteers, leukotriene B_4_ (LTB_4_) was applied epidermally to induce chemotaxis as described by Lammers et al. [100]. In this setting, Salomon demonstrated that 100 mg dapsone/day does not inhibit LTB_4_-induced chemotaxis [135]. This is in consistency with earlier findings that dapsone therapy does not induce inhibition of LTB_4_-induced chemotaxis in the skin of patients with acne [Prendiville et al., unpublished data]. Further studies with differentially activated sera again came to nothing [54, 55, 110, 131]. In conclusion, dapsone inhibits chemotaxis, but only when certain stimuli are applied. Thus, the statement that dapsone inhibits chemotaxis *per se* can no longer be sustained.

### Membrane-associated phospholipid metabolism

Cell activation produces a process of enzymatically mediated signal transduction which leads to liberation of arachidonic acid which on her part is source of biologically active mediators of the cyclooxygenase (COX)- and lipoxygenase (LOX) pathway. The influence of dapsone on choline phosphotransferase (ChPhTF) and methyltransferase I and II (MTF) was examined using PMN, lymphocytes and erythrocytes of healthy donors [122]. The investigators found a cell-specific inhibitory effect of dapsone. MTF-activity in lymphocytes and PMN was inhibited slightly by high dapsone concentrations, but not that of erythrocytes. Conversely, erythrocytes showed a significant drop of ChPhTF-activity in contrast to PMN and lymphocytes where there was no significant influence. Venom-induced inflammation following the bite of the brown recluse spider (*loxosceles* *reclusa*) has been linked to an activation of membrane phospholipids. The venom incorporates a variety of enzymes (e.g. phospholipase D, esterases, and hyaluronidase) that are believed to provoke cutaneous and even systemic effects (viscero-cutaneous loxoscelism). Using a model of cultivated human keratinocytes, venom-induced activation of membrane phospholipids could be confirmed [45]. Whether the well-known therapeutic efficacy of dapsone in the therapy of brown recluse spider bites can be attributed to the aforementioned mechanism remains speculative [165].

### Prostaglandins

Regarding the inhibition of synthesis and liberation of prostaglandins by dapsone, there is some evidence presented by Ruzicka et al. [134]. Using thin layer chromatography, the authors examined the production of prostaglandin D_2_ (PGD_2_) of mast cells that had been gathered from the plasma and peritoneal caves of Sprague–Dawley rats under the influence of dapsone (10^−2^–10^−6^ mol/L). Their experiments revealed a dose-dependent inhibition of the production of PGD_2_ (IC_50_: 1 mmol with exogenous arachidonic acid (AA)-application, 0.2–0.4 mmol with exclusively endogenous AA). Complete suppression was observed with 6 × 10^−3^ mol or 6 × 10^−2^ mol, respectively. Washing the cells following incubation with dapsone completely abolished this effect pointing to a reversible effect. Moreover, dapsone had a marked inhibitory effect on IgE-mediated antigen-stimulated production of PGD_2_ (IC50: 0.2–0.4 mmol). Interestingly, the liberation of histamine was not inhibited. As the conversion of PGH_2_ to PGD_2_ was not influenced by dapsone, the target of dapsone can be assumed to be located before the PGH-PGD-isomerase step. The authors postulate a direct inhibitory effect on COX. A similar dose-dependent inhibitory effect of dapsone on the production of PGD_2_ and 6-keto prostaglandin F_1_α (6KF_1_α) by peritoneal macrophages of mice was described by Bonney et al. [15]. Apart from mast cells and macrophages, human PMN were examined in respect to a possible inhibition of the COX pathway [6]. In contrast to clofazimine, dapsone in concentrations of 5 and 10 μg/mL inhibited both spontaneous and FMLP-induced synthesis of PGE_2_. There was no difference between the PMN of healthy donors and of leprosy patients. In summary, the inhibitory effect of dapsone on prostaglandin synthesis/liberation is beyond doubt.

### Leukotrienes

Dapsone is capable of inhibiting the generation of 5-LOX-products in PMN of healthy donors [171]. PMN were pre-treated with dapsone in different concentrations (1.6–100 μmol) subsequently by adding Ca ionophore A 23187 and subsequent incubation. Thereupon, the eicosanoids were assessed by reversed-phase high-performance liquid chromatography (RP-HPLC). Dapsone exhibited dose-dependent inhibitory activity showing 50 % inhibition at 15 μmol for LTB_4_ with 5 × 10^6^. The IC_50_ of dapsone for 5-hydroxyeicosatetraenoic acid (5-HETE) and ω-OH-LTB_4_ amounted to similar values (5-HETE: 9 μmol; ω-OH-LTB_4_: 11 μmol). In additional experiments, cells were destroyed with ultrasound and the supernatant incubated with dapsone (1.6–100 μmol) under the addition of AA. Table 2 depicts the IC_50_ calculated for LTB_4_ and 5-HETE in intact PMN and in the cell-free system. The variations indicate that dapsone also inhibits enzymatic systems beyond 5-LOX. As subsequent studies demonstrated, this might be attributed to calcium mobilization [148]. In rat mast cells, dapsone had a similar inhibitory effect on 5-LOX [134]. Maloff et al. [107] examined the effect of dapsone on LTB_4_-receptors. Using human PMN, they could demonstrate that dapsone in concentrations of 10, 50 and 100 μmol inhibits the specific binding of LTB_4_ to the receptor. A further increase of dapsone concentrations did not result in an increase of the blockade. The exact mechanism of the interruption of the ligand-receptor signal cascade is not known. In respect to an inhibition of cysteinyl leukotrienes Bonney et al. [15] examined peritoneal macrophages of male Swiss-Webster mice. Stimulation with zymosan led to a time- and dose-dependent synthesis of leukotrienes and prostaglandins. When dapsone was added in concentrations of 50, 100 and 200 μg/mL, this caused a dose-dependent suppression of LTC_4_ synthesis. The other two peptido-leukotrienes were not examined. The inhibitory effect of dapsone on LTC_4_ production could account for the observation that dapsone possesses corticosteroide-sparing effects in asthma patients For instance, Berlow et al. [10] could verify that adjuvant therapy with dapsone (2 × 100 mg/day) reduced the monthly prednisolone dose from 428 to 82 mg (*p* < 0.2) in patients with chronic, cortisone-dependent asthma. Again, the exact mechanism of this cortisone-sparing effect is not known.

**Table 2:** IC_50_ of dapsone for LTB_4_ and 5-HETE in a cell-free system (5-LOX) and in intact PMN

IC_50_ (μM)		
	Cell free (5-LOX) (5 × 10^6^ PMN)	Intakt PMN (5 × 10^6^ PMN)
LTB_4_	35.0 ± 4.3	15.0 ± 5.6
5-HETE	59.0 ± 5.1	9.0 ± 7.1

Data represent the mean ± SEM of at least three separate experiments

### Interleukin 8

Interleukin 8 (IL-8), a member of the CXC-chemokine family, purportedly plays an important role, especially in neutrophil-mediated inflammation, but has also been shown to affect other cell species as well (e.g. lymphocytes, monocytes, hematopoietic progenitor cells). Signal transduction is mediated via CXCR1 and CXCR2 receptors and is involved in viral infections and tumor progression [117]. Following observations that ROS markedly influence the regulation of IL-8 [44] studies were initiated that examined the effect of dapsone on IL 8 in different settings [44, 80]. In an in vitro model of human whole blood, dapsone in therapeutic concentrations was shown to exert a suppressive effect on lipopolysaccharide (LPS)- induced IL-8 levels [13], an observation that was confirmed by several study groups. Amongst others, dapsone suppresses bullous-pemphigoid-IgG-induced IL-8-release from keratinocytes at a posttranscriptional level [139] and is capable of inhibiting IL-8-mRNA expression [1]. Accordingly, dapsone blocks IL-8-induced chemotaxis [16, 65]. Furthermore, IL-8 has been shown to be of importance in neurologic diseases. It is expressed by neurons [174] and glioblastoma cells have been shown to be affluent sources of IL-8 [96]. As dapsone shows a suppressive effect on seizure disorders [103] and inhibits the growth of glioblastoma, it is discussed that these effects might be mediated by an inhibitory effect on IL-8 via targeting neutrophils [85]. Lately, the therapeutic application of dapsone in glioblastoma was the object of several studies [85]. However, with the advent of modern molecular-targeted strategies like inhibition of epidermal growth factor receptor, vascular endothelial growth factor, mammalian target of rapamycin (mTOR) and others, the value of dapsone in this field remains doubtful.

### TNF-α

Abe et al. [1] examined the influence of dapsone on TNF-α and other proinflammatory cytokines like IL-1β- and IL-8 in the context of its therapeutic use in lupus erythematosus. This group used PBMC from healthy donors which were stimulated with LPS. Dapsone induced a dose-dependent suppression of TNF-α at mRNA level. With the exception of IL-8, no influence on the levels of other cytokines was found. The effect on IL-8 resembled to that already reported above. It has to be pointed out, however, that the dapsone concentrations used in this study exceed that under therapy.

### Eosinophil peroxidase

In comparison to the effect on neutrophils, those on eosinophils have been far less studied. It is likely that overlapping mechanism play a role (e.g. inhibition of lipoxygenase, ROS production). Bozmen et al. examined the influence of dapsone on eosinophil peroxidase [19]. The inhibitory effect of the sulfone on MPO was even stronger than that in neutrophils and associated with a reduced production of toxic hypochlorous acid and other ROS. Consecutively, the effect of eosinophil peroxidase on mast cells was reduced (e.g. liberation of histamine) [142]. The inhibitory effect on eosinophil peroxidase could provide at least in part an explanation of the therapeutic efficacy of dapsone in eosinophil-mediated diseases (e.g. eosinophilic fasciitis).

### Complement system, coagulation and fibrinolytic system, cutaneous proteases

In vitro, an inhibitory effect of dapsone on the alternative way of complement activation was detected [Millikan et al., unpublished data]. On the contrary, Katz et al. could not find an influence of dapsone on the classic or alternative activation of complement in patients with dermatitis herpetiformis and in animal experiments [87]. Anecdotal reports document a normalization of initially decreased complement factors in immune complex vasculitis under therapy with dapsone [132, 167]. Certainly, these effects could be the result of an improvement of the disease per se and not necessarily be a result of a specific effect of dapsone on complement factors. Dapsone had no influence on plasminogen activator when epidermal cells were incubated with pemphigus antibodies [73]. Finally, the effect of dapsone on cutaneous proteases was found to be only minor [60].

### Lymphocyte function

Once more, results with regard to lymphocyte function are in part contradictory. Beiguelman and Pisam [9] described an inhibition of PHA-induced lymphocyte-transformation (LZT) by dapsone. In healthy donors who had taken dapsone in therapeutic doses for 7 days, a significant inhibition of PHA-stimulated LZT could be observed [140]. Other authors could confirm this observation when using other mitogens like concanavalin A and lepromin [5, 99]. The influence of dapsone on delayed type hypersensitivity was examined in a mouse model by Nagata et al. [119]. A daily clinical dose (1CD) of dapsone taken to be equivalent to that used in humans was 1.7 ng/kg of body weight. Up to threefold equivalent doses of dapsone (3CD) exerted no reduction of carrageenan-induced paw edema. On the other hand in mice alimentation with dapsone was followed by an emptying of the paracortical zone of lymph nodes [99]. Interestingly, dapsone loses its antibacterial potency against inoculated mycobacterium (*M. leprae*) in thymectomized naked mice, in contrast to mice with normal immune system [119]. In comparison with T-lymphocytes, B-lymphocytes are influenced by dapsone to a far lesser extent. In 20 patients with lepra lepromatosa who were treated with dapsone 50 mg/day over 12–24 months, there was no difference in the number of B-lymphocytes when compared with 26 untreated leprosy patients and 25 healthy controls [99]. Dapsone did not influence antibody-producing cells of BALB/c- and C57 BI/6-mice in a plaque-forming cell assay [119]. Furthermore, Thompson and Souhami could not find any changes in antibody production [150]. In contrast to that, Das et al. 1985 [unpublished data] demonstrated a dose-dependent inhibition of the synthesis of rabies—specific IgA- and IgG-antibodies by dapsone. The production of total IgA and IgG was not influenced. The authors attribute the inhibitory effect on specific B cell clones to a reduction of antigen-specific T-helper cell activity. The action of dapsone on Arthus reaction is controversial. Thompson and Souhami [150] as well as Ruzicka et al. [131] observed a dose-dependent suppressive effect of dapsone in guinea pigs and rabbits. Sulfoxone, a soluble sulfone, exerted no effect in guinea pigs with C4 deficiency [87].

Animal studies with liver and spleen immune cells (T- and B-lymphocytes stimulated with concanavalin A and LPS) revealed a dose-dependent effect of dapsone. At lower concentrations (1 μmol), dapsone stimulated B- and T-cell proliferation under the influence of both mitogens, but inhibited B-cell proliferation at higher concentrations (150 μmol). The authors claim an immunostimulating effect of dapsone in lower concentrations but an immunosuppressive in higher concentrations as general principle [90].

### Lifespan of *Caenorhabditis elegans*

**Fig. 6:**
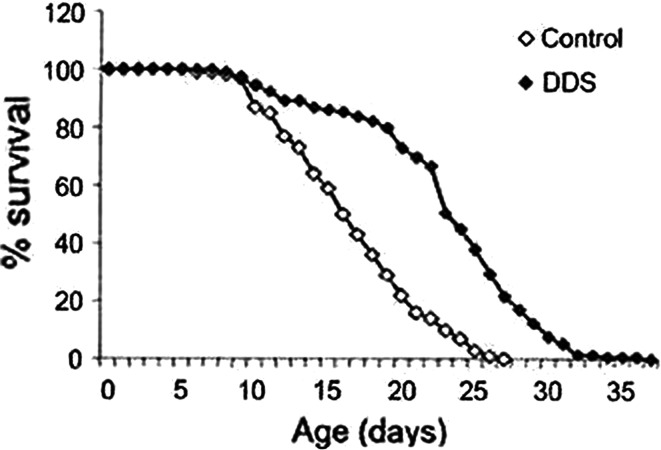
Survival curves of *Ceanorhabditis elegans* (*n* = 129) treated with dapsone (2 mmol) during their entire life time compared with worms that were untreated. (according to Cho et al. [26])

In the light of observations that—despite their disease-induced socioeconomic drawbacks—life expectancy of leprosy patients under dapsone therapy is increased, South Korean authors investigated the effect of dapsone on the lifespan in multicellular organism [26]. In their anti-aging-model, dapsone in concentrations equalling that achieved under therapy induced a marked elongation of the median lifespan of *Caenorhabditis elegans* (Fig. 6). The nematode worm is a small, relatively simple, and precisely structured organism. *C. elegans* is one of the most powerful animal models currently in use for studying the aging process and lifespan.

Dapsone delayed the induction of aging and was shown to suppress mitochondrial ROS as well as mRNA of NADPH-oxidase. Typical scavenger-enzymes like superoxide dismutase (SOD), catalase, glutathione peroxidase-/reductase were not influenced by dapsone. As already noted, this contrasts with earlier studies which had postulated a scavenger function of dapsone. More precisely, dapsone seems to inhibit the production of ROS as it has been shown by molecular studies by Cho et al. [26, 28]. Interestingly, these authors have identified muscle pyruvate kinase as the protein target of dapsone in *C. elegans*. Additional studies in long-term dapsone therapy leprosy patients using a ferric-reducing ability assay demonstrated increased antioxidative capacity in erythrocytes. These mechanisms could be reproduced in mammalian cells [26]. Experimental and clinical results thus confirm studies in human fibroblasts [27].

### Neuroprotection

Recent research revealed that dapsone can influence functions of the central nerve system [86, 120, 129, 130, 160]. More precisely, it could be shown that dapsone suppresses experimentally induced neurotoxicity [138]. In a pilot study, neuroprotective effects of dapsone could be demonstrated in stroke patients. Patients were given a single 200 mg-dose of dapsone within 12 h following the incident. A follow-up study of 60 days’ duration showed a significant reduction of National Institute of Health Stroke Scale (NIHSS) in comparison with a placebo group [118]. In order to further clarify the protective mechanism of dapsone, animal ischemia–reperfusion studies supplied evidence that dapsone exerts an inhibitory effect on lipid peroxidation, MPO, nitric oxide activity and apoptosis (annexin V, caspase 3 und 9) [46]. Again, this neuroprotective effect of dapsone was attributed to its antioxidative capacities. Anticonvulsive effects of dapsone have been described in earlier studies [4, 70, 78]. In the context of neurologic diseases, Japanese authors have reported successful therapy of steroid-dependent neuro-Sweet-disease that responded to 75 mg dapsone/day [141] (see 4.10). The neuro-Sweet-disease is characterized by multiple neurologic (e.g. meningitis, encephalitis) and well-known dermatological features (e.g. painful dull red erythematous plaques) in association with HLA-Cw1 or B54.

### Tumor growth

Following 16 days of inoculation with a continuous sixfold equivalent dose of dapsone, BALB/c-mice-myeloma cells growth showed no difference when compared with animals that had been fed with placebo [119]. In contrast, recent studies generated results pointing to an anti-cancer activity of certain dapsone derivatives [3, 12, 25, 71, 124]. At present, further research in this field is in progress.

### Human pharmacology studies

**Fig. 7:**
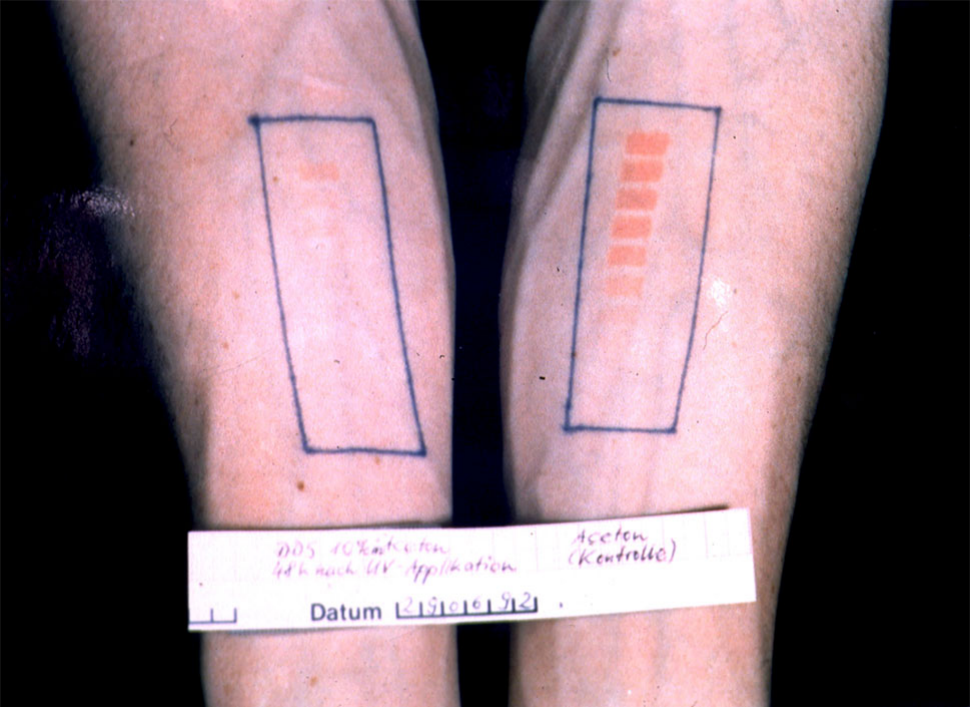
Suppression of ultraviolet (UV)-induced erythema with topical applied dapsone (1 % solved in acetone, 48 h after UV exposition) (right forearm), control: left forearm (UV source: UVB erythemal effective Hg-lamp VITALUX/OSRAM GmbH, Germany)

After topical pretreatment with dapsonemetabolites MADDS und DDS-NOH (both 1 % dissolved in acetone) for 2 weeks, 10 ng LTB_4_ was applied on skin of eight healthy volunteers. Biopsies were taken after 24 h and PMN were quantified flourometrically using elastase according to the method of Lammers et al. [100]. MADDS did not exert any inhibitory activity on PMN trafficking compared with corresponding controls and untreated area (untreated: 790 ± 450 PMN per 10 μg skin; *p* > 0.05, acetone: 840 ± 578; MADDS: 1099 ± 556), whereas DDS-NOH caused a statistical significant inhibition of PMN accumulation even exceeding the effect of the reference clobetasol-17-proprionate (CP) (DDS-NOH: 128 ± 143 PMN; CP: 86 ± 131) [172]. In contrast, the parent compound dapsone did not inhibit LTB_4_-induced chemotaxis of PMN (see section “Chemotaxis”). These in vivo results clearly demonstrate that dapsone metabolites, especially DDS-NOH, are pharmacodynamically active (Blasum et al., unpublished data). This raises the question whether attempts to increase the tolerability of dapsone therapy by trying to decrease the production of DDS-NOH might inevitably be accompanied by a loss of therapeutic efficacy (see below). In a first-proof-of-concept study with healthy volunteers there was some evidence that topical applied dapsone (1 % solved in acetone) per se may have the potential to reduce UV-induced erythema Salomon [135] (Fig. 7). In line with this pilot study are results of experiments with both, dapsone and dapsone metabolites, MADDS and DDS-NOH. [135]. Skin areas were irradiated with UVB (295 nm, two minimal erythema doses). Twenty-four hours later, UVB-induced erythema was quantified and cutaneous blood flow measured using Laser Doppler Imager. In control skin, tissue blood flow was measured to be 227 units and was significantly (*p* < 0.05) decreased by dapsone (186 U), DDH-NOH (154 U), and MADDS (195 U). UVB-induced erythema was also significantly reduced in DDS-NOH- or MADDS-treated skin when compared with controls. Thus, these experiments show that dapsone metabolites exert pharmacodynamic effects when applied topically to the skin and may at least equal dapsone in their anti-inflammatory properties. UVB erythema-suppressing activity of dapsone was subsequently confirmed by Salomon, who observed significant inhibitory effects of both topically applied (0.1–10 %) and systemically applied dapsone (100 mg/day) on UV-induced erythema in healthy volunteers having sun-reactive skin type II and III. A theoretical explanation for the observed erythema-suppressing effect of dapsone could be the drug’s inhibitory action on prostaglandins. In this context, it is noteworthy that dapsone—topically and systemically applied—unexpectedly exerted no substantial effect on anthralin- and sodium dodecylsulfate-induced erythema in healthy volunteers [135]. Dapsone metabolites were not used in these investigations.

In another human pharmacology study, dapsone was found not to possess antipsoriatic efficacy in the psoriasis plaque test [168].

## Unique characteristics of dapsone

The association of antimicrobial/antiprotozoal and antiphlogistic effects in a broad range of indications places dapsone in a unique position in the spectrum of non-steroidal antiphlogistic drugs. Currently, no other drug used in medicine possesses such a wide variety of beneficial properties as follows:

1. Combination of antimicrobial/antiprotozoal effects (utilized, e.g. in the treatment of opportunistic infections in patients with acquired immunodeficiency syndrome).
2. Safety of long-term treatment (e.g., life-long use in leprosy, long-term or chronic intermittent therapy of inflammatory dermatoses).
3. Unique powerful disease-specific antiphlogistic activities (e.g., prompt decrease of pruritus and control of skin lesions in dermatitis herpetiformis; fast amelioration of loxoscelism associated with *brown recluse spider bites*).
4. Steroid-sparing effects (e.g., long-term treatment in autoimmune blistering diseases and as an adjuvant treatment in bronchial asthma).
5. CNS-protective effects (anticonvulsive effects, reduction of stroke-associated tissue damage, inhibition of glioblastoma).

Finally, the pharmacoeconomic benefit due to the low cost of dapsone deserves to be mentioned.

## Adverse effects of dapsone

### Haematology

As already mentioned, hydroxylamines of dapsone, above all DDS-NOH, are held responsible for some of its major adverse effects including *met*-*Hb formation, haemolysis* and *agranulocytosis*. As DDS-NOH is inevitably formed during dapsone therapy, all these adverse effects are obligatory and vary only in intensity. These hematologic side effects of dapsone have long been recognized and have been reviewed by various authors [11, 101, 125, 136, 165, 173, 178]. Therefore, they will not be dealt with in detail in this survey. However, it has to be stressed that glucose-6-phosphate dehydrogenase (G6PD)-deficient patients are less susceptible to met-Hb production, but more susceptible to haemolysis. For instance, patients with G6PD-deficient erythrocytes reveal about a twofold increase in sensitivity towards sulfone-induced anaemia. Cyanosis which is usually associated with mild methaemoglobinemia may occur during dapsone treatment. Acute methaemoglobinemia occurs rarely, but may result in dyspnea, anemia, vascular collapse, and in serious courses finally in death. In patients who do not have G6PD deficiency acute methaemoglobinemia should be treated with intravenous methylene blue (1–2 mg/kg given by slow injection). The effect is generally complete within 30 min, but the procedure may need to be readministered if met-Hb reaccumulates.

### Skin

**Fig. 8:**
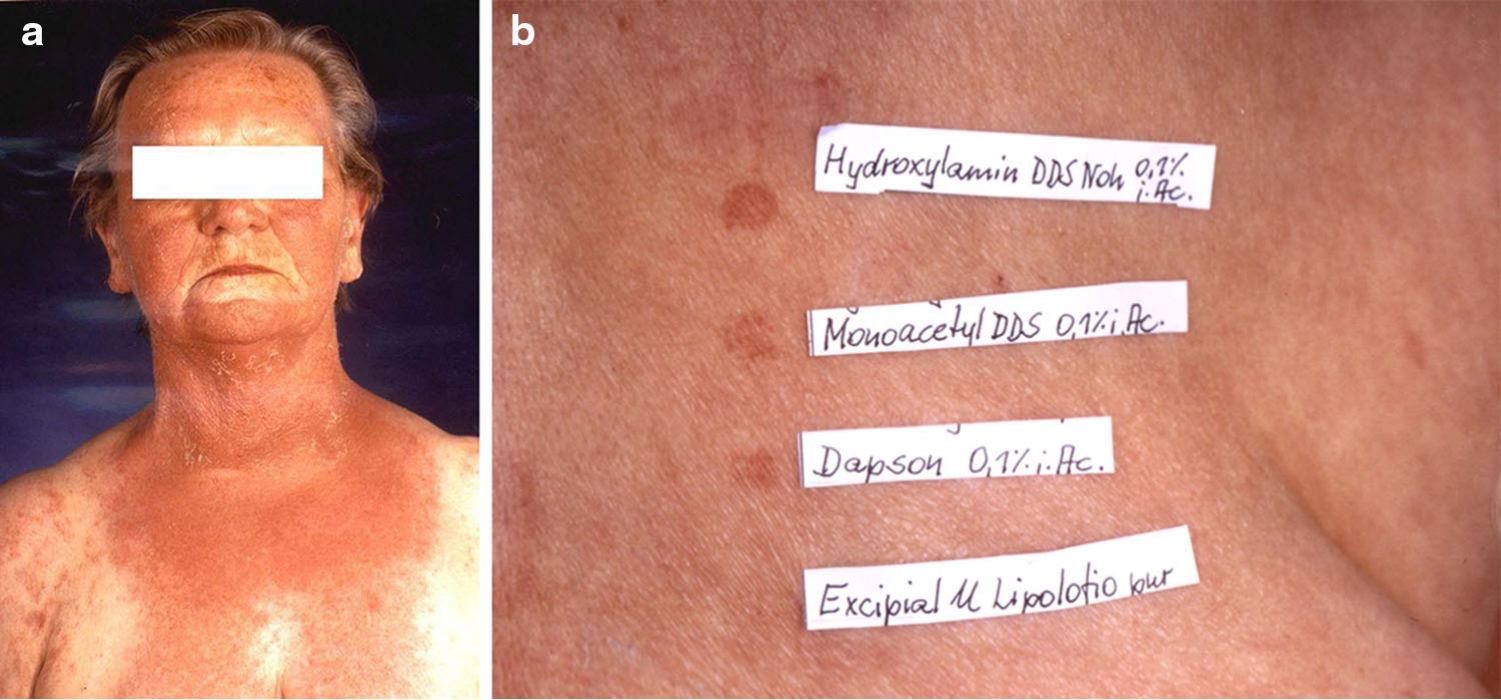
Photoallergic reaction by dapsone in a female patient with linear IgA dermatosis (**a**), Photo-patch test (5 J/cm^2^ UVA/Philips TL09) with dapsone and the two main metabolites of dapsone (**b**), (according to Stöckel et al. [145])

In respect of dermatological side effects, various skin eruptions have been described [81]. They include exfoliative dermatitis, erythema multiforme, urticaria, erythema nodosum, morbilliform and scarlatiniform exanthema and toxic epidermal necrolysis. Dapsone-induced *photosensitivity* is quite rare, not dose-dependent and might be caused not only by the parent compound dapsone but also by its metabolites (Fig. 8a, b and point 8.4.) [145].

Rash reportedly occurs in about 30–40 % of AIDS patients receiving dapsone concomitantly with trimethoprim, but less frequently in those receiving dapsone alone. Despite that observation, a substantial proportion of patients who do not tolerate co-trimoxazole are able to tolerate dapsone [41].

### Nervous system

**Fig. 9:**
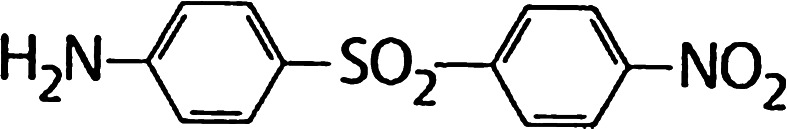
Chemical structure of dapsone hydroxylamine

**Fig. 10:**
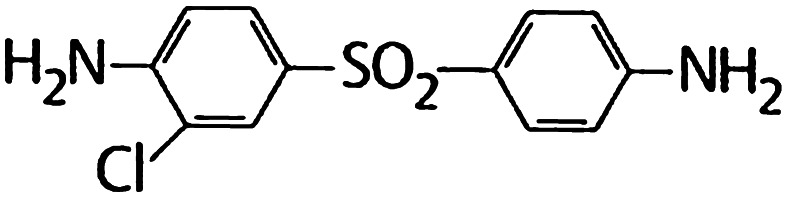
Chemical structure of chlorodapsone

**Fig. 11:**
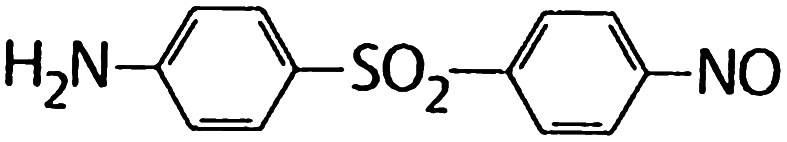
Chemical structure of a nitroderivative of dapsone

Rarely, peripheral neuropathy with primarily motor function loss has been reported in patients receiving dapsone. Recovery might occur; however, this may take many months to several years. Tragic cases of patients who had ingested dapsone with suicidal intention confirm the ability of the drug to damage the peripheral nervous system, including the optic nerve resulting in blindness [77, 91]. In any case, if any signs of neuropathy show, therapy with dapsone should be immediately stopped (Figs. 9, 10, 11).

### Gastrointestinal effects/hepatic reactions

Adverse Gastrointestinal (GI) effects including anorexia, abdominal pain, nausea and vomiting can be encountered in patients receiving dapsone. Due to their potential severity, hepatic adverse effects deserve special attention. There are three types of reactions:

1. Isolated abnormalities of liver function tests (e.g., increased bilirubin and/or liver enzymes) without evidence of hepatitis or hepatosis. If any abnormality is detected, the dosage of dapsone should be decreased or the drug should be discontinued until the source is established.
2. Prehepatic jaundice induced by haemolytic anaemia. Hyperbilirubinemia may occur more often in patients with G6PD-deficiency. All affected patients should be monitored periodically after reduction of dapsone dosage/discontinuation of treatment.
3. Toxic or cholestatic hepatitis in conjunction with hypersensitivity syndrome (HS). Hepatic coma is indeed the most frequent cause of death in the hypersensitivity syndrome [104].

### Hypersensitivity syndrome

The most recent study on HS was published in 2012. This review analyzed all published cases from 1951 (the beginning of the sulfone area) up to 2009 [104]. Of 492 reports, 114 (17 epidemiologic studies and 97 case reports) met the criteria to be included in this metaanalysis. All in all, the cases of 336 patients with HS were analyzed. Prevalence was calculated as 1.4 % (95 % confidence interval: 1.2–1.7 %). Latency of HS after initiation of dapsone treatment was ≤20 days: 24.5 %; 21–28 days: 35.0 %; 29–35 days: 20.9 %; ≥36 days: 19.6 %. Maximum latency was 20 weeks. Nearly all patients presented with a rash (91.1 %) and fever (96.9 %); 73.7 % showed lymphadenopathy. Hepatic dysfunction could be detected in the majority of cases, its severity ranging from abnormal liver tests over hepatosplenomegaly and jaundice to hepatic coma. About half of the patients demonstrated haematologic changes (leukocytosis 56.6 %, eosinophilia 43.8 %). Relevant clinical features of HS are summarized in Table 3. Following withdrawal of dapsone (and in most cases therapy with steroids), the majority of patients (82.3 %) recovered, but nearly 10 % had fatal outcome, hepatic coma being the most frequent cause of death. As expected, early discontinuation of dapsone was associated with a better prognosis. On the other hand, mucosal involvement, hepatitis, older age and concomitant diseases which occurred predominantly in third-world countries were all associated with a higher risk of fatal outcome. Considering the widespread use of dapsone and the paucity of reports, and in line with this results of the metaanalysis HS to dapsone is not a rare adverse effect. It occurs in more than 1 % of all cases [104].

**Table 3:** Clinical features of hypersensitivity reaction of dapsone (HS)

	*n* Total (*n*-334)	%
Latency (days)		
≤20	40	24.5
21 ≤ 28	57	35.0
29 ≤ 35	34	20.9
≥36	32	19.6
Complete HS^a^	149	61.1
Fever	277	96.9
Lymphadenopathy	196	73.7
Hepatitis	239	81.0
Skin symptoms	274	91.9
Exanthema/erythema	155	57.4
Erythroderma	36	13.3
Rash	79	29.3
Mycosal involvement	53	42.1
Concomitant symptoms	149	89.02
Leukocytosis	79	56.6
Anaemia	102	55.7
Eosiniphilia	78	43.8
Dapsone cessatione		
Immediately after HS onset	85	33.9
Delayed to HS onset	48	19.1
Timepoint unspecified	118	47.0
Systemic steroid therapy	167	82.3

^a^Presence of all four cardinal symptoms

Some other adverse effects of dapsone with hitherto unknown mechanisms have been recognized. Fortunately, most of them are really extremely rare (e.g. albuminuria, insomnia, psychosis, changes of electrolytes, atrioventricular block) [41, 165, 177]. Some of these may be partially attributed to anaemia and/or methaemoglobinaemia.

## Clinical use of dapsone

### Indications

Due to its antimycobacterial effects, dapsone is a component of rifampicin-based multiple drug regimes for the treatment of multibacillar and paucibacillary leprosy. Besides rifampicin, the WHO most notably recommends combinations with clofazimine [41, 67]. The antiprotozoal effect of dapsone is used in primary prophylaxis and prevention of recurrence in AIDS patients with *pneumocystis jiroveci* pneumonia and toxoplasmosis.

As anti-inflammatory agent, dapsone is employed primarily to treat chronic skin diseases characterized by an accumulation of neutrophils and/or eosinophils (Table 4). During the past years, dapsone has been introduced additionally into the therapy of several other dermatoses, mostly in combination with other drugs, namely topical or systemic steroids (Table 5). In the literature there is a huge number of case reports, clinical studies and epidemiological analyses representing beneficial effects of dapsone in such dermatological entities, collectively termed as sulfone-sensitives dermatoses. Despite a large body of evidence the subsequent enumeration is indicating only a small number of relevant references [21, 30, 31, 56, 62, 65, 68, 74, 82–84, 98, 126, 133, 143, 153, 164, 175, 179]. Finally, there are some conditions in which dapsone was reported anecdotically as being useful, but initial enthusiasm has been tempered with subsequent controversial or contradictory results and/or a total lack of properly controlled trials showing efficacy. At present, the use of dapsone in these entities can thus be advocated only in totally recalcitrant or refractory cases (see review [173]).

**Table 4:** Dapsone as first choice of treatment in chronic inflammatory dermatoses

Acropustulosis infantilis [21, 165]
Dermatitis herpetiformis Duhring [41, 56, 87, 144, 165, 178, 179]
Erythema elevatum et diutinum [65, 164, 165, 178]
IgA pemphigus [84, 178]
Linear IgA dermatosis [39, 101, 145, 151, 164, 178]
Prurigo pigmentosa [115, 165]
Subcorneal pustular dermatosis Sneddon–Wilkinson [30, 143, 164, 165, 178]

**Table 5:** Dapsone used in dermatologic entities as an adjunctive treatment modality

Brown recluse spider bite (loxoscelism) [164, 165, 178]
Bullous pemphigoid [68, 84, 126, 139, 153, 164, 165, 178]
Chronic-idiopathic urticaria/delayed pressure urticaria^a^
Cutaneous lupus erythematosus [41, 132, 133, 164, 165, 173]
Eosinophilic folliculitis (Ofuji’s disease) [173]
Leukocytoclastic vasculitis/urticaria vasculitis [62, 113, 165]
Lichen ruber pemphigoides [31]
Mucous membrane pemphigoid [74, 98]
Pemphigus vulgaris [68, 83, 84, 98, 165, 178]
Pyoderma gangrenosum [30, 164, 165]
Reccurent neutrophilic dermatosis of the dorsal hands [173]
Relapsing polychondritis [164, 165, 173, 178]
Sweet’s syndrome [141, 178]

^a^There are some case reports reporting a successful treatment of patients with chronic-idiopathic urticaria and delayed pressure urticaria vor [58, 66, 108]. Recently, dapsone was also investigated in retrospective or randomized non-blinded trials [14, 24, 42, 53, 75]. The results are confirming beneficial effects for dapsone. The German S3-guideline for treatment of chronic urticaria, therefore, recommends dapsone amongst cyclosporine, histamine_2_-receptor antagonist, omalizumab and systemics steroids as additional drug of treatment level 4 [180]

In recent years, dapsone has been employed in a variety of non-dermatological diseases. Haar et al. [69] compared the efficacy of dapsone with hydroxychloroquine and a combination of both in *rheumatoid arthritis* (RA). Dapsone was equally potent as the antimalarial. More than 10 studies have since then proved the efficacy of dapsone in RA [165]. Certainly, in the face of the introduction of biologics (e.g. TNF-α antagonists, rituximab and januskinase inhibitors like tofacitinib), the therapeutic armamentarium has been broadened to an extent that leaves dapsone a dark horse in this indication. For *eosinophilic fasciitis*, there are impressive case reports about the efficacy of dapsone [142]. Dapsone has been used in several clinical studies in patients with *immune thrombocytopenia* (*ITP*) in which the disease was refractory against standard therapy [40, 51, 52, 176]. Surprisingly, dapsone showed compelling efficacy both in adults and in children [40]. This has led to a discussion among haematologists whether dapsone should be classified as second-line therapy for patients with ITP. In *cortisone*-*dependent asthma bronchiale* patients, co-medication with dapsone allowed a reduction of steroid dose. The application of dapsone in *glioblastoma* and *seizures* has already been dealt with above (see point 4.16. and Table 6).

**Table 6:** Dapsone used in non-dermatological diseases

Rheumatoid arthritis
Eosinophilic fasciitis
Immune thrombocytopenia
Stroke
Asthma bronchiale
Seizure disorder
Glioblastoma

With these diseases, dapsone has made its way into the field of non-dermatological disorders. However, many scientific questions have not yet been answered and its definitive status remains to be elucidated.

One topical *gel formulation* of 5 % dapsone is available on the US market for treating *acne vulgaris*. Results of two large-scale studies (dapsone: *n* = 1506; vehicle: *n* = 1504) show a marginal to moderate reduction of non-inflammatory lesions compared with placebo after a 12-week treatment period (mean change: 32 % dapsone vs 42 % vehicle) [48]. For treatment of acne in adults, adolescents and children 12 years of age or older, a thin layer of dapsone gel should be applied to the cleansed affected areas. Combinations with topical benzoyl peroxide may result in temporary local yellow or orange discoloration of the skin and facial hair [41].

### Dosage of dapsone in chronic inflammatory dermatoses

The dosage of dapsone in nearly all sulfone-sensitive disorders must be individually titrated to determine the minimum daily dose that effectively controls symptoms. Typically, initial dosages in adults range from 50 to 100 mg/day. If the treatment goal is not achieved after 4–6 weeks, a higher dosage may be tried (150–300 mg/day). Administration of higher doses depends on tolerability and the results of laboratory monitoring. When a favourable response is attained, the dosage should then be reduced to the minimum that maintains a satisfactory clinical state.

For administration in children, commercially available tablets of dapsone can be crushed and dissolved, for example, in strawberry syrup. Studies evaluating bioavailability of dapsone after administration of this preparation have not been published. For some indications in childhood, such as infantile acropustulosis or eosinophilic folliculitis, a daily dosage of 2 mg per kilogram of body weight is recommended. Therapy with this dosage, or 4 mg/kg weekly, results in concentrations equivalent to those reached in adults receiving 100 mg/day. In the treatment of immune thrombocytopenia in children, dapsone was used in a similar dose of about 2 mg/kg body weight [40].

For topical treatment of acne a twice-daily application is recommended.

## Conclusions for treating patients with dapsone

### Contraindications, baseline-monitoring and follow-up controls

Dapsone is *contraindicated* in patients who are allergic to the drug. It should not be administered to patients with severe anaemia. Dapsone must be used *with caution* in the following conditions:

- Glucose-6-phosphate dehydrogenase deficiency
- Met-Hb-reductase deficiency
- Severe hepatopathy
- Cardiac insufficiency/heart failure
- Pulmonary diseases
- Co-medication with met-Hb-inducing drugs or compounds, respectively

Before initiation of dapsone therapy, patients must undergo a careful clinical evaluation that includes a complete history and physical examination. Routine laboratory checks as listed in Table 7 are recommended. Follow-up visits should include a thorough history to determine adverse effects and incorporate asking for signs of neuropathy. Laboratory tests include a complete blood count with differential and reticulocytes count at least every 2 weeks for the first 3–6 months, and then every 2–4 months. Liver and renal function tests and urinalysis should be performed monthly in the first 3–6 months and then every 2–4 months. Special caution when treating patients with dapsone must be considered in those who are receiving or have been exposed to other drugs or agents that are capable of inducing met-Hb production or haemolysis. In patients with pre-existing anaemia the exact cause of the condition should be clarified by haematologist. For treating patients with dapsone there is no threshold value of haemoglobin. The treatment initiation is based on a range of individual factors (e.g. age, profession, co-medication, daily activities, pre-existing pulmonary and/or cardiac diseases). After initiation of dapsone therapy, evaluation of met-Hb levels should be carefully addressed. Different time points are of relevance:

**Table 7:** Laboratory evaluation before initiation of dapsone therapy

Complete blood count
Bilirubin
Alanine aminotransferase
Aspartate aminotransferase
Gamma-glutamyl transferase
Creatinine
Met-Heamoglobin
Glucose-6-phosphate dehydrogenase
Serologic test for hepatitis
Urinalysis

1. Met-Hb generation after a single dapsone dose reaches its maximum after approximately 6 h and, therefore, undergoes a time-dependent process. In the early treatment phase met-Hb should be determined 4–6 h after ingestion of dapsone, because the maximum level of met-Hb in peripheral blood is indicative of real cardiopulmonary risk. For this reason, dapsone intake should be recommended in the evening in patients with activity during the day.
2. Approximately 14 days after initiation of treatment, determination of met-Hb will estimate the level *under steady*-*state conditions* and may also allow evaluation of patients adherence to therapy.
3. Other met-Hb determinations must be considered at any time if the patient`s condition has changed (e.g., increased dapsone dosage; clinical complaints, co-medication with other drugs, starting of smoking, use of pump water in agricultural regions with potential content of nitrites/anilines).

Met-Hb levels do not need to be checked if clinical conditions of the patient remain stable.

### Strategies to reduce hematologic adverse effects

*N*-hydroxylation of dapsone via the 3A-family of isoenzymes or CYP 2E1 of the hepatic cytochrome P-450 system leads to the production of DDS-NOH, which has been made responsible for haematologic side effects [35–37, 79, 111, 155]. Against this background, considerable efforts have been made to increase dapsone tolerance. Cytochrome p-450 inhibitors like ketoconazole and cimetidine reduced dapsone-associated formation of met-Hb [32–34, 128]. Co-administration of cimetidine in dosages of 3 × 400 mg/day reduced met-Hb-levels by 25–30 % in vivo, whereupon this effect surprisingly decreased after 3 months. Coleman recommended to routinely co-administer cimetidine at higher dapsone doses (e.g. 200 mg/day and more) [38]. It has to be borne in mind, however, that DDS-NOH possesses anti-inflammatory effects on its own right. Theoretically, suppressing its production might reduce the therapeutic effects of the parent compound dapsone. This question has not yet been addressed in studies. As another strategy, various antioxidants like ascorbic acid and vitamin E have been examined. Neither a dose of 1000 mg ascorbic acid/day [89, 127, 146] nor 800 mg vitamin E had a significant effect on dapsone-induced haemolysis [127]. Only simultaneous application of ascorbic acid and vitamin E exerted an inhibitory effect on met-Hb formation [127]. The reason for these conflicting results might be attributable to the design of the studies which at that time were not GCP-based (no randomization, blinding or placebo controls). For daily practice, in cases of pronounced haematologic adverse effects, co-administration of cimetidine during the first weeks of treatment can be recommended if there are no alternative treatment modalities.

### Potential risk: concomitant use of local anaesthetics

It is well-known that co-medication with other inductors of methaemoglobinaemia as phenacetine and sulphonamides is risky in patients under therapy with dapsone. However, the risk associated with concomitant application of local anaesthetics like benzocaine or lidocaine is often neglected [61]. Cases have been reported where application of local anaesthetics of the amid or ester type as a spray or cream resulted in severe methaemoglobinaemia up to 35 % manifesting right away during surgical procedures. Thus, patients should always carry a dapsone pass and be instructed to present it to their doctors. In elective surgery, dapsone should be stopped a few days earlier. As a precaution, met-Hb levels should be determined before surgery.

### Prevention of photosensitivity

Dapsone is able to suppress UV-induced erythema both when applied orally and topically [166]. However, allergic photosensitivity to dapsone cannot be excluded; but apparently, this is an extremely rare event. So far, less than 20 cases have been reported. As photo-patch tests revealed, the characteristic sulfone group of the parent compound and dapsone metabolites is responsible for the allergic reaction [145]. In view of the suppressive effects of dapsone on UV-induced erythema, it might be wise to advise at least skin type I patients to reckon with increased photosensitivity when dapsone therapy is stopped.

### Cross-allergy between dapsone and sulfonamides

Several lines of evidence suggest that there is no obligatory cross-reactivity between sulfonamides and other sulfonamide-nonantibiotic drugs including dapsone [147]. FDA-approved label for dapsone states that sulfonamide allergy does “not include as a contraindication for dapsone use”. Currently, absence of cross-reactivity between dapsone and sulfonamides was confirmed and ascribed to the differential chemical structure of these two drugs [162]. In conclusion, dapsone is not contraindicated in patients with sulfonamide-allergy.

### Pregnancy

Animal reproduction studies with dapsone have not been conducted. Dapsone has been tested in rats and rabbits with concentrations 500- to 800-fold higher than therapeutic levels, when referenced to AUC-value in female patients under therapy with a 5 % dapsone gel. In these studies, dapsone was associated with embryocidal effects [41]. There are, however, no documented noxious effects of dapsone when used in pregnancy [94]. Especially publications about the use of dapsone in the therapy of leprosy suggest that dapsone can be used safely in pregnancy. Multiple case reports about the use of dapsone in a variety of diseases support the assumption of innocuousness. [39, 50, 76, 97, 105, 106, 109, 154, 158]. In any case, the risk of not treating the mother must be weighed up against the risk for the fetus. Dapsone is labelled by US FDA as drug of pregnancy category C. Patients should be informed about the lack of secured data.

In terms of drug interactions and management of acute toxicity of dapsone, the reader is referred to several reviews on this topic [7, 35, 41, 165].

## Conclusion

Dapsone has been studied by far most frequently in vitro—under strictly defined experimental settings. Overall, results of these experiments paint a picture of *possible* mechanisms of action of dapsone. In addition, many data from human in vivo studies are available including experience in dapsone-sensitive diseases. Nevertheless, we are still far from really understanding dapsone with its differing, in part contradictory facets in most non-infectious diseases. As a result, there are many questions in respect of the mechanisms of action of dapsone that remain to be addressed:

1. In past years much progress in developing molecular-based methods has substantially led to new insights into mechanisms of action of immunosuppressive and/or immunomodulating remedies including biologics. Therefore, to get more detailed information about the mechanisms of action of dapsone, this panel of investigations should be used with dapsone (e.g., pharmacogenetics) in concentrations comparable with therapeutic conditions.
2. Most of the experimental and clinical studies have been conducted with the parent compound dapsone. Hitherto, the relevance of dapsone metabolites has not been clarified sufficiently. Modern molecular targeted analyses can certainly help to rectify these deficits. This aspect has not yet been addressed at all in the scientific literature.
3. Experimental clues to a preventive effect of dapsone on neurotoxicity require further clarification, as the proposed mechanisms leave room for speculations.
4. Observations of a positive effect of dapsone on lifespan in the *Caenorhabditis elegans* model raise new questions concerning the inhibition of ageing effects of the sulfone in mammalian cells and organisms.
5. Finally, dapsone has experienced a renaissance as reference molecule in organic chemistry as efforts are made to synthesize biologically active dapsone analogues like sulfoximine [3, 12, 25, 124]. In this context the definition of the anti-cancer activity of dapsone derivatives currently is of special interest.

Despite that bundle of unanswered questions, dapsone remains an indispensable drug for dermatology and a scientifically fascinating remedy. Discovering dapsone congeners which exhibit the positive therapeutic properties of the original drug but lack unwanted side effects appears desirable.

## Abbreviations

AA: Arachidonic acid
AB: Antibody
AIDS: Acquired immunodeficiency syndrome
AIP: α-1-Protease inhibitor
ALAT: Alanine-aminotransferase
ASAT: Aspartate-aminotransferase
AUC: Area under curve
*C. elegans*: *Caenorhabditis elegans*
CBC: Complete blood count
ChPhTF: Cholinephosphotransferase
CNS: Central nervous system
C5a: Complement component 5a
COX: Cyclooxygenase
CP: Clobetasol-17-propionate
CXCR: CXC chemokine receptor
CYP 450/2E1: Cytochrome P450/2E1
DDS: 4,4′-Diaminodiphenylsulfone
DDS-NOH: Dapsone hydroxylamine
DTH: Delayed type hypersensitivity
EAS: Endotoxin activated autologous serum
EGF/EGFR: Epidermal growth factor/epidermal growth factor receptor
FACS: Fluorescence-activated cell sorter
FDA: Food and drug administration
FMLP: *N*-formyl-methionyl-leucyl-phenylalanine
G6PD: Glucose-6-phosphate dehydrogenase
5-HETE: 5-Hydroxyeicosatetraenoic acid
HS: Hypersensitivity syndrome
IC_50_: Half-maximal inhibitory concentration
IgA/G/E: Immunoglobulin A/G/E
IL-8: Interleukin 8
IL-1β: Interleukin 1β
INFγ: Interferon γ
ITP: Idiopathic thrombocytopenic purpura
KC: Keratinocyte
6KF1α: 6-Keto-prostaglandin F_1_α
LDCF: Leukocyte-derived chemotactic factor
LE: Lupus erythematosus
LFA: Leukocyte function antigen
5-LOX: 5-Lipoxygenase
LTB_4 _/LTC_4_: Leukotriene B_4_/C_4_
LPS: Lipopolysaccharide
LC: Lymphocyte
MADDS: Monoacetyl dapsone
MEST: Mouse ear swelling test
met-Hb: Methemoglobin
MPO: Myeloperoxidase
mRNA: Messenger ribonucleic acid
mTOR: Mammalian target of rapamycin
NADPH: Reduced nicotinamide adenine dinucleotide phosphate
NSAID: Non-steroidal anti-inflammatory drug
NIHSS: National Institute of Health Stroke Scale
PAF: Platelet-activating factor
PBMC: Peripheral blood mononuclear cell
PGD_2_: Prostaglandin D_2_
PHA: Phytohemagglutinin
PMA: Phorbol myristate acetate
PMN: Polymorphonuclear leukocytes
RA: Rheumatoid arthritis
ROS: Reactive oxygen species
SOD: Superoxide dismutase
TNFα: Tumor necrosis factor α
UDP: Uridine diphosphate
UVA/UVB: Ultraviolet A/B
VEGF/VEGFR: Vascular endothelial growth factor/VEGF receptor
